# Day to Day Blood Pressure Variability Associated With Cerebral Arterial Dilation and White Matter Hyperintensity

**DOI:** 10.1161/HYPERTENSIONAHA.122.19269

**Published:** 2022-05-03

**Authors:** Boyu Zhang, Yajing Huo, Zidong Yang, Huihui Lv, Yilin Wang, Jianfeng Feng, Yan Han, He Wang

**Affiliations:** Institute of Science and Technology for Brain-Inspired Intelligence (B.Z., Z.Y., J.F., H.W.), Fudan University, Shanghai, China.; Department of Neurology, Zhongshan Hospital (H.W.), Fudan University, Shanghai, China.; Human Phenome Institute (H.W.), Fudan University, Shanghai, China.; Key Laboratory of Computational Neuroscience and Brain-Inspired Intelligence (Fudan University), Ministry of Education, China (B.Z., Z.Y., J.F., H.W.).; Department of Neurology, Yueyang Hospital of Integrated Traditional Chinese and Western Medicine, Shanghai University of Traditional Chinese Medicine, Shanghai, China (Y. Huo, H.L., Y. Han).; Georgetown Preparatory School, Washington DC (Y.W.).

**Keywords:** angiography, blood pressure, cerebral small vessel disease, dementia, hypertension

## Abstract

Previous studies suggested blood pressure variability (BPV) might help reveal interactions between blood pressure fluctuation and white matter lesions, and the impact of elevated BPV on white matter hyperintensity (WMH) or cerebral arterial dilation is unclear.

**Methods:**

This retrospective observational study involved 2634 stroke-free individuals (68.6±11.1 years, 50.3% female), who underwent magnetic resonance imaging and magnetic resonance angiography scans, from a single center in Shanghai, China. Measurements for variability of blood pressure were made based on 7 days blood pressure recordings. WMHs were quantified from T2-FLAIR images and further classified as periventricular WMH or deep WMH. M1 segment of middle cerebral artery dilation was assessed from magnetic resonance angiography images. General linear model was used to examine the associations.

**Results:**

Both increased systolic and diastolic BPV were associated with increased WMH volume (systolic: *β*=0.02 [95% CI, 0.004–0.03], *P*=0.01; diastolic: *β*=0.05 [95% CI, 0.03–0.08], *P*<0.001). Only periventricular WMH was associated with BPV (systolic: *β*=0.02 [95% CI, 0.005–0.04], *P*=0.01; diastolic: *β*=0.06 [95% CI, 0.04–0.09], *P*<0.001). MCA dilation was found in 125 individuals (4.75%). Systolic BPV was associated with MCA dilation only in the hypertensive individuals (*β*=0.11 [95% CI, 0.06–0.17], *P*<0.001). Increased WMH volume was found associated with dilated MCA (*β*=0.17 [95% CI, 0.11–0.23], *P*<0.001).

**Conclusions:**

Increased BPV might be one of the pathophysiological phenomena involving in the small vessel disease independent of hypertension. Increased BPV might independently contribute to intracranial arterial dilation. Management of BPV might be a target to preserve cerebrovascular wellness.

**Novelty and Relevance**

**What Is New?**

High blood pressure variability is linked to white matter hyperintensity and intracranial arterial dilation independent of hypertension.

**What Is Relevant?**

Even in nonhypertensive individuals, diastolic blood pressure variability was associated with increased white matter lesion volume. Moreover, excessive variability in systolic blood pressure was associated with intracranial arterial dilation only in the hypertensive individuals.

**Clinical/Pathophysiological Implications?**

Besides hypertension, management of blood pressure variability might be a target to preserve cerebrovascular wellness.

Hypertension affects around one-third of adults worldwide.^1^ Being a leading cerebrovascular risk, hypertension has been reported to have deleterious effects on the cerebral vessels, resulting in cerebrovascular morbidities including stroke and cerebral small vessel disease (CSVD).^2,3^ Episodic hypertension, based on the average of blood pressure (BP) measurements upon hospital admission, is commonly used to define hypertension.^4^ Yet, such definition may, very often, not reliably reflect the real BP of the patients.^5^ Not to mention it also disregards fluctuations in the BP levels.^6^

However, as an organ with high blood flow, the brain is vulnerable, not only to elevated BP, but also to rapid changes in BP.^7^ Excessive fluctuations in BP would affect vessels supplying the deep white matter or periventricular regions due to their anatomic features.^8^ Previous studies have suggested that extreme BP variability (BPV) over time could lead to higher risks of dementia and stroke.^9,10^ Moreover, extreme BPV was reported to impact large vessels, increasing arterial stiffness which predisposes risks for future stroke.^11^ Intracranial arterial dolichoectasia (IADE), distinct from intracranial atherosclerotic stenosis and characterized as an excessive increase in length or diameter of at least one intracranial artery, is a dilative arteriopathy.^12,13^ IADE has a different pathophysiological changes of arterial walls compared with intracranial atherosclerotic stenosis and, therefore, may be influenced by different risk factors commonly reported to contribute to intracranial atherosclerotic stenosis.^13^ However, only aging was consistently found to be correlated with increased cerebral arterial diameters in previous studies.^14,15^ The association between BPV and IADE has less been reported.

Increased BPV, meanwhile, could also affect vessels of smaller sizes. CSVD, characterized by several neuroimaging features, including recent small subcortical infarcts, cerebral microbleeds, lacunes, and white matter hyperintensities (WMHs), results in significantly increased risk of stroke and is found involved in approximately half of the dementia cases.^16,17^ As the most commonly observed clinical feature of CSVD, WMHs have been under extensive investigation, and their relationship with hypertension has frequently been reported.^18,19^ The relationship between WMHs and BPV, however, has been largely debated.^20^

To investigate the complex nature of lesions of both large and small cerebral vessels and their relations with extreme BP fluctuations, we monitored the BP of subjects for 7 consecutive days and analyzed the day-to-day BPV in a large group of patients free of stroke.^21^ We hypothesize that increased BPV would result in lesions of the both large and small cerebral vessels, manifested as larger WMH lesion volume and more severe IADE. WMHs were classified as the periventricular WMH (PWMH) or deep WMH (DWMH) to further study the differential effects of BPV on anatomically distinctive vessels, and the impact of BPV on WMH spatial distribution was reported.

## Materials and Methods

### Data Availability Statement

The data that support the findings of this study are available from the corresponding author upon reasonable request.

### Participants

This retrospective, observational study involves data from the Yueyang Hospital of Integrated Traditional Chinese and Western Medicine, Shanghai, China. Patients hospitalized in the department of neurology and underwent brain magnetic resonance imaging (MRI) and magnetic resonance angiography (MRA) between January 2017 and June 2021 were included as the candidates. Subjects with acute stroke, dementia, carotid, or cerebral artery stenosis were excluded. Cerebral artery stenosis was defined as any degree stenosis in at least one of the following arteries: intracranial carotid artery, middle cerebral artery (MCA), anterior artery, intracranial segment of vertebral artery, basilar artery, and posterior cerebral artery.^22^ The subjects involved in this study were mostly elderly individuals with traditional cerebrovascular risk factors. The main reasons for their visits were due to headache, dizziness, sleep disorders, palpitation, gait disturbances, memory loss, or concentration problems. Patients with CT-confirmed signs of CSVD were admitted for hospitalization, and conventional BP and heart rate monitoring and brain MRI were performed for further evaluation of CSVD. Neuropsychological test, sleep monitoring, and gait analysis were performed only for part of the subjects. The study uses only previously collected data, with no additional tests or interventions undertaken on any participants and thus would inflict no impact on patient care or the outcome. The Ethics Committee of Yueyang Hospital of Integrated Traditional Chinese and Western Medicine, Shanghai University of Traditional Chinese Medicine approved the current study (Number: 2020-060).

### Data Collection

Demographic characteristics, personal and family medical history, and lifestyle risk factors were collected in the clinic. Cigarette use, alcohol consumption, hypertension, diabetes, hyperlipidemia, history of cardiovascular disease (coronary artery disease, atrial fibrillation, heart failure, or heart valve disease) were assessed. Hypertension was defined as self-reported hypertension, treatment with antihypertensive agents, systolic BP ≥140 mm Hg, or diastolic BP ≥90 mm Hg. Venous blood samples were taken by certified nurses between 7 am and 8 am after overnight fasting and used for the determination of lipid profile (including total cholesterol, HDL [high-density lipoprotein] cholesterol, triglycerides), creatinine, glucose, and glycated hemoglobin.

BP was measured after the subject rested at least 5 minutes in a seated position. An appropriately sized cuff was placed on the dominant forearm arranged at the horizontal level of the fourth intercostal space at the sternum and BP was measured using a validated digital electronic tensiometer OMRON (OMRON Corp., Kyoto, Japan). In the following days, BP was taken from the same arm, at a similar time. BPV was calculated based on first 7 consecutive measurements after admission. Three commonly used BPV parameters were assessed including within-individual SD (mm Hg), coefficient of variation (CoV; SD/mean×100, %) and variation independent of mean (VIM, mm Hg). VIM was calculated as the SD divided by the within-individual mean to the power *p* and multiplied the average value of SBP in the cohort to the power *p*, which is obtained by fitting a curve of SD against the within-individual mean SBP.^11^

### Brain MRI and Analysis

Individuals underwent brain MRI and MRA scan on one 3.0T MR scanner (Philips Ingenia, Philips Healthcare, Best, the Netherlands). The MRI protocol included T1-weighted structural imaging, T2 FLAIR, and time-of-flight MRA with more protocol details in the Supplemental Material.

WMH lesions were automatically segmented from the T2 FLAIR images. The total WMH, PWMH, and DWMH lesion volume were obtained after registration of T2 FLAIR images to the Montreal Neurological Institute brain template.^23^ The presence of WMH in the basal ganglia region was defined as at least 0.01 mL of WMH in this region. Voxel-wise regressions were performed to investigate the impact of BPV on WMH spatial distributions,^24^ with more details in the Supplemental Material.

The M1 segment of the MCA was manually quantified from the vascular segmentation on MRA images (Figure S1). The mean diameter of each vascular segment was assessed. The sum of mean diameter of left and right M1 segment was used. Presence of MCA dilation was defined as the within-individual diameter ≥ mean diameter in the cohort + 2 SD in the cohort.^13^ The internal carotid artery and basilar artery were not included in the analysis as their origin could not be clearly defined in the MRA images. For more detailed information for the MRI analysis, please see Methods in the Supplemental Material.

### Statistical Analysis

Multivariable linear regression analysis was used to assess the association of BPV with cerebral artery dilation and WMH. Due to the skewed distribution of WMH lesion volume, logarithmic transformation was applied. General linear model with logistic link function and binomial distribution was used for the binary response variable. The results of normally distributed continuous variables were presented as mean and SD or as median and interquartile range. For categorical variables, frequencies and proportions were presented. Two-sample *t* test or χ^2^ test was applied to evaluate group differences according to requirement. All tests were 2-sided, and a *P* value of <0.05 was considered significant. All statistical analysis was undertaken using Matlab (version 2021a, MathWorks, Natick, MA).

## Results

### Individual Characteristics

**Figure 1.**
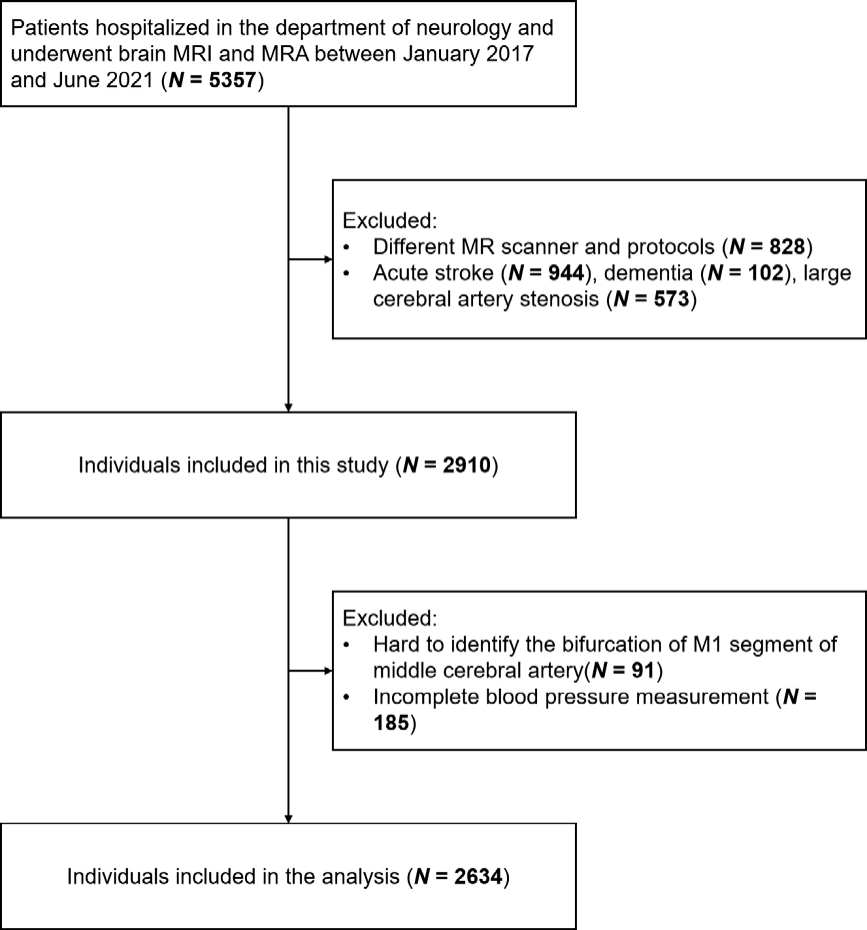
**Flow chart of enrollment in the current study.** MRA indicates magnetic resonance angiography; and MRI, magnetic resonance imaging.

The individual characteristics are presented in Table 1. Figure 1 summarizes the enrollment process of the current study. A total of 2910 individuals were initially included; of these, 91 individuals for whom the bifurcations of MCA could not be identified and 185 individuals with incomplete BP measurement were excluded. Therefore, 2634 individuals were included in the subsequent analysis. The mean (SD) age was 68.6 (11.1) years and 1325 individuals were female (50.3%).

**Table 1.**
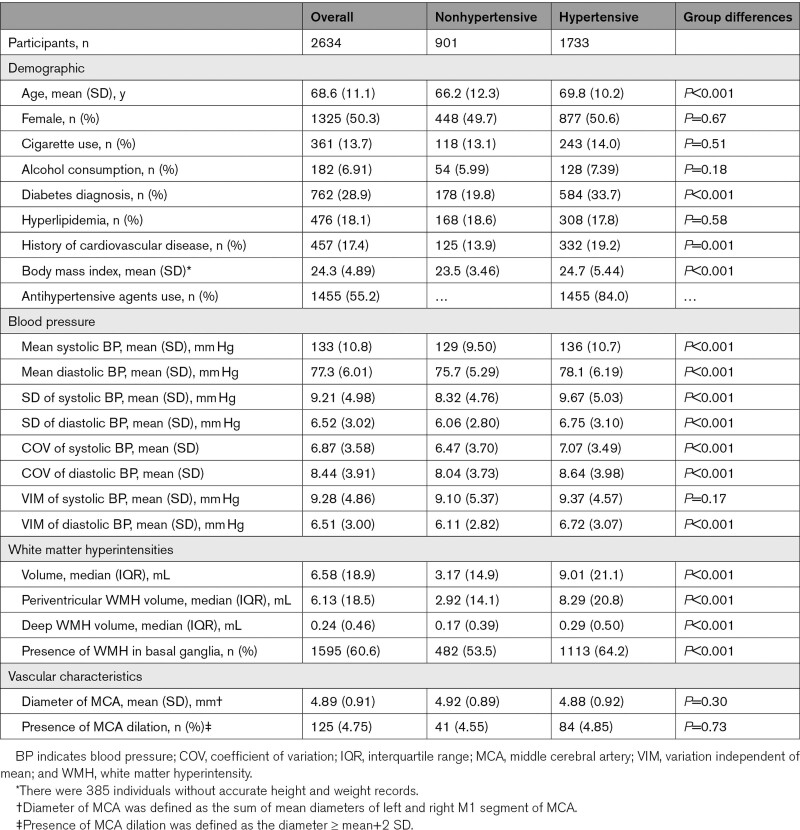
Participants Characteristics

Age, sex, and hypertension were found to be associated with BPV in bivariable models (Table S1). In multivariable models, age remained associated with both systolic and diastolic BPV, and sex was associated with systolic BPV.

### Association Between BPV and WMH

In general, higher SD, CoV, and VIM were found to be associated with increased total WMH volume, after adjusting for age, sex, and risk factors for both systolic (systolic SD: β=0.03 [95% CI, 0.02–0.05], *P*<0.001; systolic CoV: β=0.04 [95% CI, 0.02–0.06], *P*<0.001; systolic VIM: β=0.02 [95% CI, 0.004–0.03], *P*=0.01) and diastolic BPV (diastolic SD: β=0.06 [95% CI, 0.04–0.08], *P*<0.001; diastolic CoV: β=0.04 [95% CI, 0.02–0.06], *P*<0.001; diastolic VIM: β=0.05 [95% CI, 0.03–0.08], *P*<0.001), as shown in the Table 2. When considering PWMHs and DWMHs, BPV was significantly associated with only PWMH volume. Only SD was found to be associated with DWMH volume after adjusting for age and sex (systolic SD: β=0.03 [95% CI, 0.004–0.05], *P*=0.02; diastolic SD: β=0.04 [95% CI, 0.01–0.08], *P*=0.01), and the association remained for diastolic SD after adjusted for risk factors (β=0.03 [95% CI, 0.002–0.07], *P*=0.038).

**Table 2.**
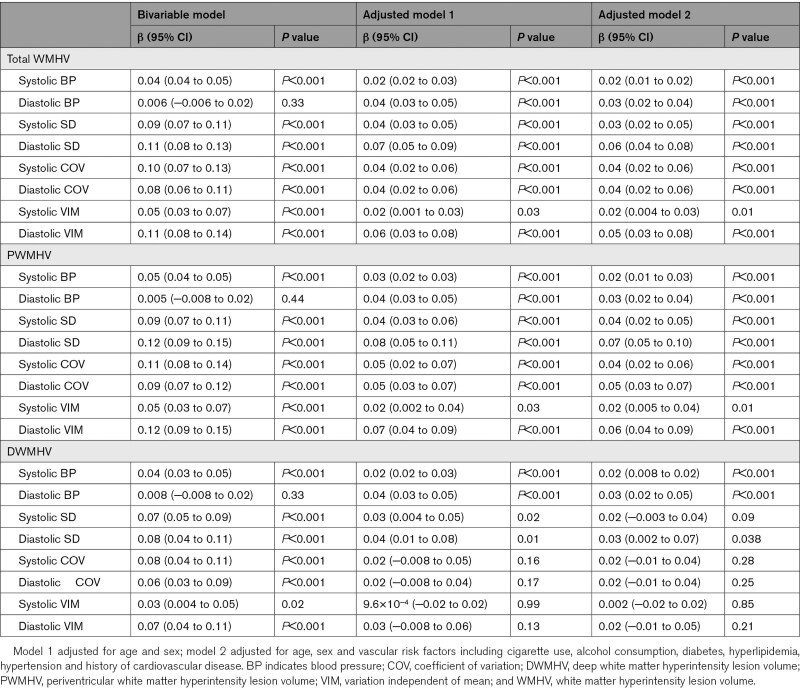
Association Between Blood Pressure Variability and White Matter Hyperintensity

These associations were further examined in hypertensive and nonhypertensive individuals (Tables S2 and S3). In the hypertensive individuals, these associations remained, whereas in the nonhypertensive individuals, only diastolic BPV was associated with WMH volume. Nevertheless, no significant interaction between BPV and hypertension status on WMH volume was observed (Table S4).

**Figure 2.**
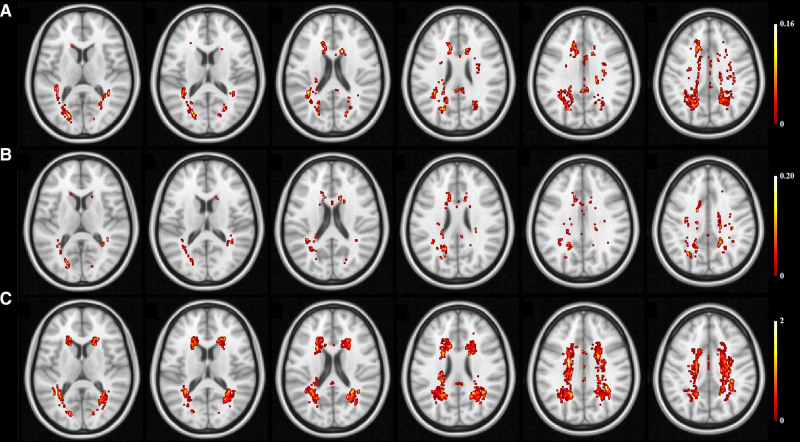
**Blood pressure variability and hypertension associated distribution of white matter hyperintensity. A**, Variation independent of mean (VIM) of systolic blood pressure. **B**, VIM of diastolic blood pressure. **C**, hypertension status.

Figure 2 shows BPV and hypertension associated distribution of WMH. WMHs associated with SBP VIM and DBP VIM mainly were found in the anterior and posterior horns of the lateral ventricles and the centrum semiovale, and most were on the posterior white matter. Hypertension or high BP associated WMHs were noted in the area around the anterior horns of the lateral ventricles, the posterior horns of the ventricles and the centrum semiovale. Other BPV features associated distribution of WMH was presented in the Figure S2.

### Association Between BPV and Cerebral Arterial Dilation

Association between BPV and cerebral arterial dilation was presented in Table 3. Only systolic BPV measures were associated with the diameter of MCA (systolic SD: β=0.01 [95% CI, 0.002–0.02], *P*=0.02; systolic CoV: β=0.02 [95% CI, 0.003–0.03], *P*=0.01; systolic VIM: β=0.01 [95% CI, 0.002–0.02], *P*=0.02). After adjusted for age, sex and risk factors, higher systolic SD (systolic SD: β=0.01 [95% CI, 0.001–0.02], *P*=0.03; systolic CoV: β=0.01 [95% CI, 0.002–0.03], *P*=0.02; systolic VIM: β=0.01 [95% CI, 7.4×10^−4^ to 0.02], *P*=0.03) was still associated with increased MCA diameter. Meanwhile, presence of MCA dilation was associated with only three systolic BPV measures (systolic SD: β=0.09 [95% CI, 0.05–0.13], *P*<0.001; systolic CoV: β=0.12 [95% CI, 0.06–0.18], *P*<0.001; systolic VIM: β=0.07 [95% CI, 0.03–0.12], *P*=0.001), and such associations remained after adjusted for age, sex and risk factors (systolic SD: β=0.08 [95% CI, 0.04–0.12], *P*<0.001; systolic CoV: β=0.11 [95% CI, 0.05–0.17], *P*<0.001; systolic VIM: β=0.07 [95% CI, 0.03–0.12], *P*=0.001). In the subgroup with BMI information, after adjusted for BMI and all risk factors, presence of MCA dilation still associated with elevated BPV, while no significant association between MCA diameter and BPV was found as shown in the Table S5.

**Table 3.**
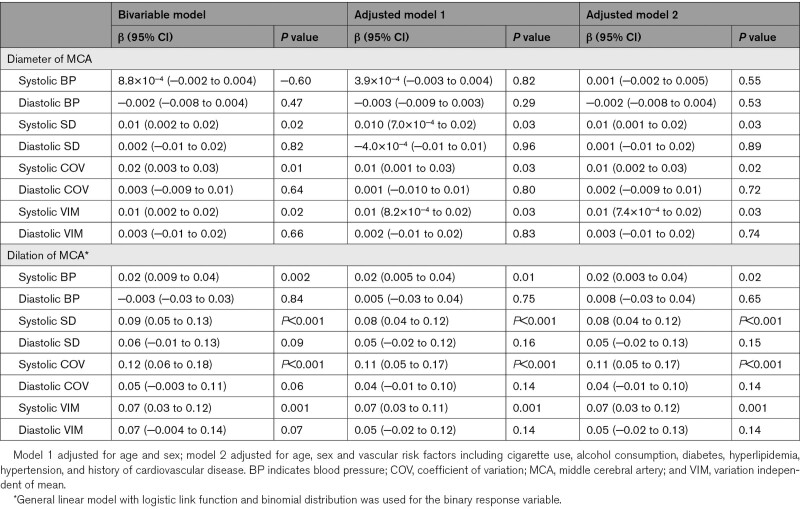
Association Between Blood Pressure Variability and Cerebral Arterial Dilation

As shown in the Table S6, in hypertensive individuals, increased systolic VIM was associated with increased diameter of MCA (β=0.02 [95% CI, 0.004–0.03], *P*=0.01) after adjusted for age, sex, and risk factors. No significant association between BPV measures and cerebral arterial dilation was found in nonhypertensive individuals (Table S7), and no significant interactions were observed (Table S8).

### Cerebral Arterial Dilation and WMH

The relationships between cerebral arterial dilation and WMH were further examined as shown in Table 4. Total WMH was associated with MCA diameter before (β=0.22 [95% CI, 0.14–0.29], *P*<0.001) and after adjusted for age, sex, and risk factors (β=0.17 [95% CI, 0.11–0.23], *P*<0.001). Higher total WMH volume was also found in individuals with dilated MCA (β=0.96 [95% CI, 0.62–1.3], *P*<0.001). Such patterns were similarly observed for PWMH, but not DWMH.. Only MCA diameter was associated with DWMH volume after adjusted for age, sex and risk factors (β=0.11 [95% CI, 0.02–0.20], *P*=0.01). In a more focal view, the presence of WMH in the basal ganglia region was significantly associated with increased MCA diameter (β=0.28 [95% CI, 0.18–0.37], *P*<0.001) and MCA dilation (β=1.0 [95% CI, 0.55–1.44], *P*<0.001).

**Table 4.**
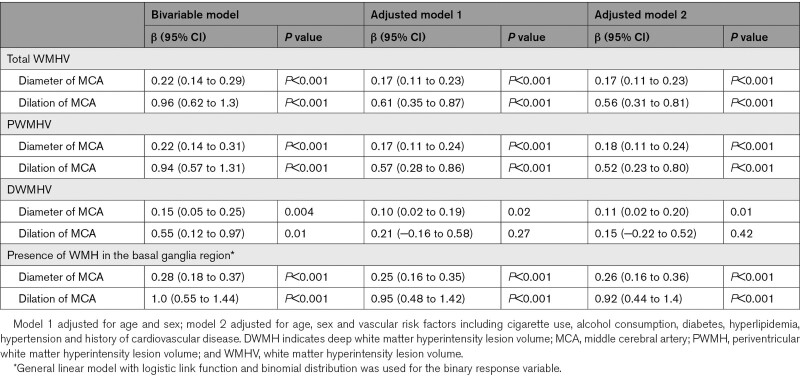
Association Between Cerebral Arterial Dilation and White Matter Hyperintensity

## Discussion

In this study, BPV indices including CoV, SD, and VIM were calculated based on consecutive measurements of BP over 7 days. CoV allows us to see how great the dispersion is around the mean, as larger CoV values indicate greater variability in BP. VIM, however, was used to help assess the associations of BPV with WMH and arterial dilation irrespect of the mean BP levels. Consequently, significant associations of VIM with several indices of vascular lesions identified in this study suggest large fluctuations in BP alone would increase the liability of the cerebrovasculature to damages. The current study provides preliminary data for future prospective studies investigating the driving forces for spatially distinct clusters of WM lesions and also suggests more active investigations on stabilizing BP for hypertensive individuals as a possible means to preserve the soundness of the cerebrovasculature should be carried out.

### BPV and WMH

High BP during mid-life is widely recognized as a modifiable risk for late-life cerebrovascular disease.^25,26^ To date, the association between high BP and CSVD in elderly individuals has been well established in cross-sectional and longitudinal studies.^27,28^ In addition to higher BP, large variations in BP from consecutive measures may also contribute to CSVD.^29–31^ Nonetheless, such relationship between BPV and WMH is still largely debated. Zhou et al^20^ suggested that long-term or short-term SD of BP are not associated with WMH features measured, and similarly, others reported no association between 24-hour SD of BP and the presence of WMH.^32^ In this study, increases in all three short-term measurements of BPV in systolic or diastolic BP were found to be associated with increased WMH lesion volume independent of hypertension status, and the results are the most pronounced in hypertensive patients. Such results may suggest that increased mean BP may increase the vulnerability of the vascular system to large fluctuations in BP.

Various BPV measurements have been used in previous studies which might be the reason for the inconsistencies found in the reported association between BPV and cerebral lesions. For example, Zhou et al^20^ used SD based on seven days home BP measurements, while Filomena et al^30^ used several BPV features including SD, CoV, and weighted-SD based on 24 hours BP monitoring. Another possible reason for these inconsistent results was the differences in the measurement of WMH. In this study, after dividing the WMHs into PWMHs and DWMHs, elevated BPV, as indicated by all 3 BPV indices used, was only found associated with higher PWMH volume. Moreover, the spatial distribution pattern of WMH associated with elevated BPV is distinct from that associated with hypertension, suggesting the spatial distinct clusters of WMHs might be implicated in different pathological processes. PWMH was associated with mean arterial pressure, since the periventricular region were supplied by long straight arteries with very few branches, resulting in greater pressure transmitted directly to the small resistance vessels.^8,33^ On the contrary, the small arterioles of the cortex usually branch off long arteries with numerous branches, which resulted in a drop in BP.^8^ Variations in BP would, thus, expected to have diminished effects on the white matter. The heterogeneous nature of WMHs could probably underpin the discrepancies found in previous studies. Future studies should be cautious in evaluating WMH, while more attention is needed to explore other mechanisms associated with DWMH.

### BPV and Cerebral Artery Dilation

The cerebral artery dilation discussed in the current study is the one of the common used imaging diagnostic criteria of IADE.^12,13^ The prevalence of MCA dilation in the current study was nearly 5%, similar to the prevalence reported in the stroke-free population (5.4%) and lower than that in patients with stroke (12%).^34,35^

In this study, systolic BPV was associated with intracranial arterial dilation. In previous studies, risk factors for IADE have been mainly investigated in patient with stroke.^13^ Aging, hypertension, and a history of myocardial infarction have been associated with IADE.^34^ In a stroke-free cohort study, only aging was found to be associated with IADE.^35^ Nevertheless, BPV has been associated with the functions of vessel wall, including endothelial function and smooth muscle function,^36^ which may be one of the reasons for the link between BPV and IADE. Furthermore, BPV was found to be associated with the prevalence of MCA dilation only in hypertensive individuals. This result suggested that elevated BPV might be more likely to induce intracranial vasodilation in hypertensive individuals. Since the arterial stiffness is associated with hypertension,^37^ fragile vessels walls might be more vulnerable to elevated BPV, leading to the development of excessive arterial dilation. However, the mechanism of MCA dilation in normotensive individuals remains unclear.

### Cerebral Artery Dilation and WMH

Previous studies have reported the association between carotid dilation and CSVD.^38,39^ In this study, except for carotid dilation, intracranial arterial diameters were assessed. Increasement of MCA diameter was associated with increased WMH lesion volume, adding the additional knowledge that intracranial arterial dilation was associated not only with the presence of WMH but also with the severity of WMH. There were at least 2 possible explanations for this finding.

First, proximal vessels might undergo compensatory dilation when distal small vessel disease happened. In the experimental study, cerebral hypoperfusion due to unilateral common carotid artery occlusion is gradually restored over several months by an increase in diameter of the contralateral internal carotid artery.^40^ The association of increased diameter of the carotid artery with CSVD is also thought to be a compensatory mechanism to counteract the increase in stiffness and thickness of the arterial wall to maintain normal arterial compliance.^38^

Second, proximal vascular dilatation might lead to increased distal BP, which results in the fragile distal vessels being more vulnerable to the impact of BP. The cerebral circulation is thought to be susceptible to pressure damage, since the torrential circulation receives less vascular resistance.^41^ According to the Poiseuille’s law, the pressure drop is inversely proportional to the fourth power of the lumen radius. As a result, the dilated proximal vessels inevitably have a reduced partial pressure capacity, and thus elevating the BP in the distal vessels would lead to the disruption of small vessels. Moreover, it has been suggested that elevated BP may affect CSVD by reshape the large intracranial arteries.^42^

Nevertheless, it is more likely that the above 2 processes interact with each other, forming a vicious circle, which promotes the growth of WMH. A similar vicious cycle related to morphological modifications between large and small arteries caused by hypertension has been reported previously.^43^ Future longitudinal studies could help to distinguish the sequence of these two processes, and thus allowing for different interventions to reduce damage to the cerebrovascular system.

### Limitation

There are several limitations to be addressed. First, day-to-day BPV measures can only be obtained from hospitalized subjects in this current study, since home BP measuring requires training of the subjects and the quality/credibility of data cannot be gauranteed. Further investigations are needed to determine the relationships of BPV over longer periods or 24-hour BPV with WMH and IADE. Second, the assessments of cerebral arterial dilation were based on internal diameter of the vessels. It is not yet available to direct assess modifications in the vessel wall from our current MRA scans. The use of additional imaging modalities and techniques will facilitate direct analysis of the intracranial vascular wall. Third, the configuration of the circle of Willis was not included in the analysis. The effect of the circle of Willis on the cerebral arterial dilation still needs further studies. Fourth, causal relationships between BPV and the vasculopathies could not be established in this study due to the retrospective design and observational nature. Since increased BPV might be a result of disturbed cerebral autoregulation caused by damaged vascular services. Further longitudinal studies will help distinguish the sequence of these 2 pathological alterations, and thus allowing for different interventions to reduce damage to the cerebrovascular system. Although a large sample was drawn (2634), most of them were elder Chinese residing in Shanghai, with two-thirds of them being hypertensive. As a result, our findings were not pronounced in the normotensive subpopulation and should also be interpreted with caution. Future studies involving more normotensive individuals are required to establish the association between BPV and cerebral vasculopathy.

### Perspectives

This study contributes to elucidating the impact of BPV on the cerebral vasculature. Currently, higher BP have been associated with increased CSVD. This study indicated that variation in BP, independent with BP level, was associated with both large cerebral arterial modification and small vessel disease, suggesting that elevated BPV might be one of the common pathophysiological phenomena involving in these cerebral vasculopathy. Cautions, though, were warranted since our study, limited by the retrospective design, was incable of establishing a causal relationship between excessive BPV and cerebral vasculopathy. Future longitudinal studies investigating the temporal sequence of cerebral vascular lesions and BPV changes would be required for better clinical relevance.

## Article Information

### Acknowledgments

We are greatly thankful to all the members in our research group at Fudan University and Yueyang Hospital who helped to accomplish the study.

### Sources of Funding

This work was supported by the National Key R&D Program of China (No. 2018YFC1312900), National Natural Science Foundation of China (No. 81971583), Shanghai Natural Science Foundation (No. 20ZR1406400), Shanghai Municipal Science and Technology Major Project (No.2017SHZDZX01, No.2018SHZDZX01) and ZJLab.

### Disclosures

None.

### Supplemental Materials

Detailed Methods

Tables S1–S8

Figures S1 and S2

References 23,24,42

## Supplementary Material


