# Supplementary material for: Day to Day Blood Pressure Variability Associated With Cerebral Arterial Dilation and White Matter Hyperintensity
Source: Hypertension. 2022 May 3;79(7):1455–65. doi: 10.1161/HYPERTENSIONAHA.122.19269 (PMC9172904; doi:10.1161/HYPERTENSIONAHA.122.19269)

Day to day blood pressure variability associated with cerebral arterial dilation and white matter hyperintensity

Boyu Zhang[1,2], Yajing Huo[3], Zidong Yang[1,2], Huihui Lv[3], Yilin Wang[4], Jianfeng Feng[1,2], Yan Han[3], He Wang[1,2,5,6]

Author affiliations:

[1]Institute of Science and Technology for Brain-Inspired Intelligence, Fudan University, Shanghai, China

[2]Key Laboratory of Computational Neuroscience and Brain-Inspired Intelligence (Fudan University), Ministry of Education, China

[3]Department of Neurology, Yueyang Hospital of Integrated Traditional Chinese and Western Medicine, Shanghai University of Traditional Chinese Medicine, Shanghai, Shanghai, China

[4]Georgetown Preparatory School, Washington DC, USA

[5]Department of Neurology, Zhongshan Hospital, Fudan University, Shanghai, China

[6]Human Phenome Institute, Fudan University, Shanghai, China

Supplementary Material Content

This supplementary material includes:

Supplemental Methods

Supplementary Table S1: Association between blood pressure variability and vascular risk factors

Supplementary Table S2: Association between blood pressure variability and white matter hyperintensity in hypertension individuals

Supplementary Table S3: Association between blood pressure variability and white matter hyperintensity in non-hypertension individuals

Supplementary Table S4: Interaction between blood pressure variability and hypertension on the association of white matter hyperintensity

Supplementary Table S5. Association between blood pressure variability and cerebral arterial dilation adjusted for BMI.

Supplementary Table S6: Association between blood pressure variability and cerebral arterial dilation in hypertension individuals

Supplementary Table S7: Association between blood pressure variability and cerebral arterial dilation in non-hypertension individuals

Supplementary Table S8: Interaction between blood pressure variability and hypertension on the association of cerebral arterial dilation

Supplementary Figure S1: Scheme of manual determination of cerebral vessels and Representative manually drawn points

Supplementary Figure S2: Blood pressure and variability associated distribution of white matter hyperintensity.

Supplemental Methods

MRI protocol

The MRI protocol included T1-weigthed structural imaging (TE=2.3ms, TR=250ms, flip angle=75°, matrix size=512×512×18, voxel size=0.45mm×0.45mm×6mm), T2-weighted FLAIR (TE=120ms, TR=7000ms, flip angle=90°, matrix size=384×384×18, voxel size=0.6mm×0.6mm×6mm) and time-of-flight MRA (TE=3.5ms, TR=23ms, flip angle=18°, matrix size=560×560×112, voxel size=0.375mm×0.375mm×0.8mm).

White matter hyperintensity analysis

WMH lesions were segmented automatically by the lesion prediction algorithm as implemented in the LST toolbox (www.statistical-modelling.de/lst.html) for SPM (https://www.fil.ion.ucl.ac.uk/spm/). Only T2 FLAIR images were used to detect the WMH lesions. For each individual, T2-FLAIR images were registered to the T1-weighted images using an affine transformation and the T1-weighted images were registered to the MNI brain atlas with voxel size=1mm×1mm×1mm by non-linear transformation. Then, two transformations were concatenated to transform the T2-FLAIR images and corresponding WMH lesions images into the reference space. WMH lesions were divided into periventricular WMH or deep WMH according to the distance from the lateral ventricles (>10 mm was considered to be deep WMH).[23] Presence of WMH in the basal ganglia region was defined as at least 0.01 mL of WMH in the basal ganglia region.

Voxel-wise regressions were carried out to investigate the impact of BPV on WMH spatial distributions. The general linear model with the logistic link function and the binomial distribution of response variable was fitted at each voxel.[23] The presence or absence of WMH was treated as the dependent variable, and hypertension status and blood pressure variability (BPV) were treated as the independent variable adjusted for age and sex. These processes were performed on MATLAB (version 2021a, MathWorks, Natick, Massachusetts, USA).

Vascular characteristics

Cerebral vessel segmentation was completed on MRA images using the previously described methods.[42] The vessels were then reconstructed into the 3D space to allow interactive manual selection of start and end points of the first segment (M1) of the middle cerebral artery (MCA). The morphological features and the centerline information of the MCA was calculated based on the segments between each pair of start and end points. Details were described blow.

The vascular bifurcations are the segment where two child vessels diverge from a single parent vessel. However, to analyze each of the vascular branches, the bifurcations are indeed represented as single centerline points and used to set the start and end points of a certain vascular branch. The following steps were performed to quantify the features of the MCA:

1) Individuals with scans where the bifurcations of anterior cerebral artery (start/endpoints of the MCA) are not visible are excluded. To quantify features of MCA, bifurcations need to be selected from the reconstructed vessels.
2) The segmented vessels were reconstructed into the 3D space. Using the Skeleton

3D toolbox, vessels then are represented as “centerline points” (Figure S1). According to the number of centerline points in their 26-neighbors, each point was assigned as endpoint, link points (Figure S1, gray dots) and bifurcation points (Figure S1, red dots).

3) Two trained individuals manually selected the bifurcation points of MCA to ensure the accuracy of the results from step 2. To do so, the reconstructed vessels in 3D space was then loaded into MeVisLab to allow interactive manual selection of the bifurcations (red dots, Figure S1). Figure S1 B shows the representative points.
4) The results were then visually inspected, bifurcation points from step 2 were only regarded correct if they match those manually selected bifurcations.
5) Then MCA was represented as all the link points between two bifurcation points.

The diameter of MCA was calculated based on the centerline points. Mean diameter was defined as the mean of the diameter calculated at each centerline link point. The sum of mean diameter of left and right M1 segment was used.

Table S1. Association between blood pressure variability and vascular risk factors

SBP, systolic blood pressure. DBP, diastolic blood pressure. SD, standard deviation. COV, coefficient of variation. VIM, variation independent of mean. CVD, cardiovascular disease.

	SBP		DBP		SBP_SD		DBP_SD		SBP_COV		DBP_COV		SBP_VIM		DBP_VIM	
	β	p value	β	p value	β	p value	β	p value	β	p value	β	p value	β	p value	β	p value
Bivariable																
Age	0.15	p<0.001	-0.11	p<0.001	0.05	p<0.001	0.01	0.01	0.03	p<0.001	0.03	p<0.001	0.03	p<0.001	0.02	p<0.001
Sex	-0.95	0.02	-1.88	p<0.001	-0.45	0.003	-0.33	p<0.001	-0.31	0.004	-0.23	0.047	-0.35	0.01	-0.2	0.03
Cigarette use	1.08	0.06	1.66	p<0.001	0.32	0.13	0.28	0.03	0.20	0.18	0.20	0.23	0.20	0.32	0.17	0.19
Alcohol consumption	2.93	p<0.001	2.66	p<0.001	0.44	0.12	0.51	0.004	0.20	0.33	0.39	0.09	0.05	0.86	0.32	0.06
Diabetes	3.09	p<0.001	0.08	0.75	0.47	0.004	0.06	0.54	0.22	0.06	0.08	0.52	0.05	0.72	0.06	0.52
Hyperlipidemia	0.22	0.67	1.06	p<0.001	-0.16	0.40	0.08	0.49	-0.12	0.38	-0.02	0.88	-0.17	0.36	-0.006	0.96
Hypertension	6.57	p<0.001	2.38	p<0.001	0.85	p<0.001	0.38	p<0.001	0.33	0.003	0.28	0.02	-0.09	0.54	0.23	0.01
History of CVD	0.53	0.31	-0.48	0.11	-0.04	0.82	0.10	0.42	-0.05	0.75	0.18	0.25	-0.09	0.62	0.13	0.27
Multivariable																
Age	0.13	p<0.001	-0.11	p<0.001	0.05	p<0.001	0.01	0.01	0.03	p<0.001	0.03	p<0.001	0.03	p<0.001	0.02	p<0.001
Sex	-0.8	0.05	-1.68	p<0.001	-0.44	0.006	-0.31	0.002	-0.31	0.008	-0.23	0.08	-0.37	0.02	-0.19	0.055
Cigarette use	0.19	0.77	-0.47	0.2	0.24	0.36	0.02	0.91	0.18	0.34	0.10	0.63	0.22	0.38	0.07	0.66
Alcohol consumption	2.57	0.002	1.38	0.003	0.24	0.47	0.37	0.07	0.05	0.83	0.33	0.21	-0.14	0.66	0.26	0.19
Diabetes	1.94	p<0.001	-0.23	0.33	0.27	0.10	-0.03	0.77	0.12	0.3	-0.007	0.96	0.02	0.89	-0.009	0.93
Hyperlipidemia	0.88	0.08	0.90	0.001	0.07	0.73	0.17	0.15	0.02	0.86	0.11	0.46	-0.03	0.89	0.10	0.41
Hypertension	5.83	p<0.001	2.81	p<0.001	0.65	p<0.001	0.34	p<0.001	0.22	0.06	0.17	0.17	-0.19	0.21	0.16	0.10
History of CVD	-0.52	0.30	-0.008	0.98	-0.32	0.10	0.05	0.70	-0.21	0.15	0.05	0.73	-0.21	0.26	0.04	0.73

Table S2. Association between blood pressure variability and white matter hyperintensity in hypertension individuals.

Model 1 adjusted for age and sex; Model 2 adjusted for age, sex and vascular risk factors including cigarette use, alcohol consumption, diabetes, hyperlipidemia and history of cardiovascular disease. BP, blood pressure. SD, standard deviation. COV, coefficient of variation. VIM, variation independent of mean. WMHV, white matter hyperintensity lesion volume. PWMH, periventricular white matter hyperintensity lesion volume. DWMH, deep white matter hyperintensity lesion volume.

	Bivariable model		Adjusted Model 1		Adjusted Model 2	
	β (95% CI)	P value	β (95% CI)	P value	β (95% CI)	P value
Total WMHV						
Systolic BP	0.02 (0.01 to 0.03)	*P*<0.001	0.01 (0.008 to 0.02)	*P*<0.001	0.01 (0.007 to 0.02)	*P*<0.001
Diastolic BP	-0.02 (-0.03 to -0.009)	0.001	0.02 (0.006 to 0.03)	0.003	0.02 (0.008 to 0.03)	0.001
Systolic SD	0.07 (0.06 to 0.09)	*P*<0.001	0.04 (0.02 to 0.06)	*P*<0.001	0.04 (0.02 to 0.05)	*P*<0.001
Diastolic SD	0.07 (0.04 to 0.10)	*P*<0.001	0.06 (0.03 to 0.08)	*P*<0.001	0.06 (0.03 to 0.08)	*P*<0.001
Systolic COV	0.10 (0.08 to 0.13)	*P*<0.001	0.05 (0.03 to 0.07)	*P*<0.001	0.05 (0.03 to 0.07)	*P*<0.001
Diastolic COV	0.07 (0.04 to 0.09)	*P*<0.001	0.04 (0.02 to 0.06)	*P*<0.001	0.04 (0.02 to 0.06)	*P*<0.001
Systolic VIM	0.07 (0.05 to 0.09)	*P*<0.001	0.03 (0.01 to 0.05)	*P*<0.001	0.03 (0.01 to 0.05)	0.001
Diastolic VIM	0.09 (0.05 to 0.12)	*P*<0.001	0.05 (0.03 to 0.08)	*P*<0.001	0.05 (0.03 to 0.08)	*P*<0.001
PWMHV						
Systolic BP	0.02 (0.01 to 0.03)	*P*<0.001	0.02 (0.008 to 0.02)	*P*<0.001	0.01 (0.007 to 0.02)	*P*<0.001
Diastolic BP	-0.02 (-0.04 to -0.01)	0.001	0.02 (0.003 to 0.03)	0.02	0.02 (0.004 to 0.03)	0.009
Systolic SD	0.08 (0.06 to 0.10)	*P*<0.001	0.04 (0.03 to 0.06)	*P*<0.001	0.04 (0.02 to 0.06)	*P*<0.001
Diastolic SD	0.08 (0.04 to 0.11)	*P*<0.001	0.06 (0.03 to 0.09)	*P*<0.001	0.07 (0.04 to 0.09)	*P*<0.001
Systolic COV	0.11 (0.08 to 0.14)	*P*<0.001	0.06 (0.03 to 0.08)	*P*<0.001	0.06 (0.03 to 0.08)	*P*<0.001
Diastolic COV	0.07 (0.05 to 0.10)	*P*<0.001	0.05 (0.02 to 0.07)	*P*<0.001	0.05 (0.02 to 0.07)	*P*<0.001
Systolic VIM	0.08 (0.05 to 0.10)	*P*<0.001	0.04 (0.02 to 0.06)	*P*<0.001	0.04 (0.02 to 0.06)	*P*<0.001
Diastolic VIM	0.10 (0.06 to 0.13)	*P*<0.001	0.06 (0.03 to 0.09)	*P*<0.001	0.06 (0.03 to 0.09)	*P*<0.001
DWMHV						
Systolic BP	0.02 (0.010 to 0.03)	*P*<0.001	0.01 (0.006 to 0.02)	0.001	0.01 (0.004 to 0.02)	0.004
Diastolic BP	-0.02 (-0.04 to -0.007)	0.005	0.02 (5.1×10^{-4} to 0.03)	0.049	0.02 (5.3×10^{-4} to 0.03)	0.043
Systolic SD	0.06 (0.04 to 0.08)	*P*<0.001	0.03 (0.006 to 0.05)	0.01	0.03 (0.005 to 0.05)	0.02
Diastolic SD	0.05 (0.009 to 0.09)	0.02	0.04 (0.002 to 0.07)	0.039	0.04 (0.001 to 0.07)	0.042
Systolic COV	0.08 (0.05 to 0.12)	*P*<0.001	0.03 (0.002 to 0.07)	0.035	0.03 (7.9×10^{-4} to 0.07)	0.045
Diastolic COV	0.05 (0.02 to 0.08)	0.003	0.02 (-0.005 to 0.05)	0.10	0.02 (-0.005 to 0.05)	0.11
Systolic VIM	0.06 (0.03 to 0.08)	*P*<0.001	0.02 (-0.006 to 0.05)	0.13	0.02 (-0.006 to 0.04)	0.14
Diastolic VIM	0.06 (0.02 to 0.10)	0.003	0.03 (-0.005 to 0.07)	0.09	0.03 (-0.006 to 0.07)	0.10

Table S3. Association between blood pressure variability and white matter hyperintensity in non-hypertension individuals.

Model 1 adjusted for age and sex; Model 2 adjusted for age, sex and vascular risk factors including cigarette use, alcohol consumption, diabetes, hyperlipidemia and history of cardiovascular disease. BP, blood pressure. SD, standard deviation. COV, coefficient of variation. VIM, variation independent of mean. WMHV, white matter hyperintensity lesion volume. PWMH, periventricular white matter hyperintensity lesion volume. DWMH, deep white matter hyperintensity lesion volume.

	Bivariable model		Adjusted Model 1		Adjusted Model 2	
	β (95% CI)	P value	β (95% CI)	P value	β (95% CI)	P value
Total WMHV						
Systolic BP	0.07 (0.06 to 0.09)	P<0.001	0.03 (0.02 to 0.04)	P<0.001	0.03 (0.01 to 0.04)	P<0.001
Diastolic BP	0.03 (-0.002 to 0.05)	0.07	0.04 (0.02 to 0.06)	P<0.001	0.04 (0.02 to 0.06)	P<0.001
Systolic SD	0.08 (0.05 to 0.12)	P<0.001	0.03 (9.2×10^{-4} to 0.06)	0.043	0.03 (-3.4×10^{-4} to 0.05)	0.053
Diastolic SD	0.14 (0.08 to 0.20)	P<0.001	0.07 (0.02 to 0.11)	0.003	0.06 (0.02 to 0.11)	0.004
Systolic COV	0.07 (0.02 to 0.12)	0.006	0.02 (-0.01 to 0.06)	0.19	0.02 (-0.01 to 0.06)	0.20
Diastolic COV	0.10 (0.05 to 0.14)	P<0.001	0.04 (0.005 to 0.07)	0.03	0.04 (0.004 to 0.07)	0.03
Systolic VIM	0.01 (-0.02 to 0.05)	0.44	0.005 (-0.02 to 0.03)	0.67	0.006 (-0.02 to 0.03)	0.63
Diastolic VIM	0.13 (0.07 to 0.19)	P<0.001	0.05 (0.008 to 0.09)	0.02	0.05 (0.007 to 0.09)	0.02
PWMHV						
Systolic BP	0.08 (0.06 to 0.09)	P<0.001	0.03 (0.02 to 0.04)	P<0.001	0.03 (0.01 to 0.04)	P<0.001
Diastolic BP	0.03 (-0.004 to 0.05)	0.09	0.04 (0.02 to 0.06)	P<0.001	0.04 (0.02 to 0.06)	0.001
Systolic SD	0.09 (0.05 to 0.13)	P<0.001	0.03 (-7.1×10^{-4} to 0.06)	0.051	0.03 (-0.002 to 0.06)	0.06
Diastolic SD	0.16 (0.10 to 0.23)	P<0.001	0.08 (0.03 to 0.13)	0.001	0.08 (0.03 to 0.13)	0.001
Systolic COV	0.08 (0.02 to 0.13)	0.006	0.03 (-0.01 to 0.06)	0.20	0.03 (-0.01 to 0.06)	0.21
Diastolic COV	0.11 (0.06 to 0.16)	P<0.001	0.05 (0.01 to 0.09)	0.007	0.05 (0.01 to 0.08)	0.008
Systolic VIM	0.02 (-0.02 to 0.05)	0.43	0.006 (-0.02 to 0.03)	0.66	0.007 (-0.02 to 0.03)	0.62
Diastolic VIM	0.15 (0.08 to 0.21)	P<0.001	0.07 (0.02 to 0.11)	0.005	0.07 (0.02 to 0.11)	0.006
DWMHV						
Systolic BP	0.07 (0.05 to 0.09)	P<0.001	0.02 (0.006 to 0.04)	0.008	0.02 (0.002 to 0.04)	0.03
Diastolic BP	0.04 (5.2×10^{-4} to 0.07)	0.047	0.05 (0.02 to 0.08)	0.001	0.05 (0.02 to 0.08)	0.001
Systolic SD	0.06 (0.01 to 0.11)	0.02	0.006 (-0.04 to 0.05)	0.78	0.004 (-0.04 to 0.05)	0.85
Diastolic SD	0.10 (0.01 to 0.18)	0.02	0.02 (-0.05 to 0.09)	0.54	0.02 (-0.05 to 0.09)	0.57
Systolic COV	0.04 (-0.02 to 0.11)	0.20	-0.004 (-0.06 to 0.05)	0.88	-0.005 (-0.06 to 0.05)	0.85
Diastolic COV	0.06 (-5.4×10^{-4} to 0.12)	0.052	-0.002 (-0.05 to 0.05)	0.95	-0.002 (-0.05 to 0.05)	0.92
Systolic VIM	-0.005 (-0.05 to 0.04)	0.83	-0.01 (-0.05 to 0.02)	0.50	-0.01 (-0.05 to 0.03)	0.53
Diastolic VIM	0.08 (0.001 to 0.16)	0.047	2.9×10^{-4} (-0.07 to 0.07)	0.99	-7.1×10^{-4} (-0.07 to 0.07)	0.98

Table S4. Interaction between blood pressure variability and hypertension on the association of white matter hyperintensity.

Model 1 adjusted for age and sex; Model 2 adjusted for age, sex and vascular risk factors including cigarette use, alcohol consumption, diabetes, hyperlipidemia and history of cardiovascular disease. HTN, hypertension. BP, blood pressure. SD, standard deviation. COV, coefficient of variation. VIM, variation independent of mean. WMHV, white matter hyperintensity lesion volume. PWMH, periventricular white matter hyperintensity lesion volume. DWMH, deep white matter hyperintensity lesion volume.

	Bivariable model		Adjusted Model 1		Adjusted Model 2	
	β (95% CI)	P value	β (95% CI)	P value	β (95% CI)	P value
Total WMHV						
HTN*Systolic BP	-0.05 (-0.07 to -0.04)	*P*<0.001	-0.02 (-0.03 to -0.008)	0.001	-0.02 (-0.03 to -0.007)	0.002
HTN*Diastolic BP	-0.05 (-0.07 to -0.02)	0.001	-0.008 (-0.03 to 0.01)	0.48	-0.005 (-0.03 to 0.02)	0.64
HTN*Systolic SD	-0.009 (-0.05 to 0.03)	0.64	-0.002 (-0.03 to 0.03)	0.91	-0.001 (-0.03 to 0.03)	0.92
HTN*Diastolic SD	-0.06 (-0.12 to 0.001)	0.054	-0.02 (-0.06 to 0.03)	0.52	-0.01 (-0.06 to 0.04)	0.63
HTN*Systolic COV	0.03 (-0.02 to 0.08)	0.22	0.01 (-0.03 to 0.05)	0.50	0.01 (-0.03 to 0.05)	0.52
HTN*Diastolic COV	-0.03 (-0.07 to 0.02)	0.30	-0.008 (-0.04 to 0.03)	0.68	-0.006 (-0.04 to 0.03)	0.76
HTN*Systolic VIM	0.06 (0.02 to 0.10)	0.002	0.02 (-0.008 to 0.05)	0.16	0.02 (-0.009 to 0.05)	0.18
HTN*Diastolic VIM	-0.04 (-0.10 to 0.03)	0.25	-0.01 (-0.06 to 0.04)	0.67	-0.008 (-0.06 to 0.04)	0.75
PWMHV						
HTN*Systolic BP	-0.06 (-0.07 to -0.04)	*P*<0.001	-0.02 (-0.03 to -0.008)	0.002	-0.02 (-0.03 to -0.007)	0.003
HTN*Diastolic BP	-0.05 (-0.08 to -0.02)	0.001	-0.01 (-0.03 to 0.01)	0.39	-0.007 (-0.03 to 0.02)	0.54
HTN*Systolic SD	-0.010 (-0.05 to 0.03)	0.66	-0.002 (-0.04 to 0.03)	0.91	-0.001 (-0.04 to 0.03)	0.94
HTN*Diastolic SD	-0.08 (-0.14 to -0.007)	0.03	-0.03 (-0.08 to 0.03)	0.30	-0.02 (-0.08 to 0.03)	0.38
HTN*Systolic COV	0.03 (-0.02 to 0.09)	0.24	0.02 (-0.03 to 0.06)	0.52	0.01 (-0.03 to 0.06)	0.52
HTN*Diastolic COV	-0.03 (-0.09 to 0.02)	0.20	-0.02 (-0.06 to 0.03)	0.45	-0.01 (-0.06 to 0.03)	0.52
HTN*Systolic VIM	0.06 (0.02 to 0.10)	0.003	0.02 (-0.01 to 0.06)	0.18	0.02 (-0.01 to 0.06)	0.19
HTN*Diastolic VIM	-0.05 (-0.12 to 0.02)	0.16	-0.02 (-0.08 to 0.03)	0.43	-0.02 (-0.07 to 0.04)	0.50
DWMHV						
HTN*Systolic BP	-0.05 (-0.07 to -0.03)	*P*<0.001	-0.02 (-0.04 to -0.002)	0.03	-0.02 (-0.03 to -6.9×10^{-4})	0.042
HTN*Diastolic BP	-0.06 (-0.09 to -0.02)	0.001	-0.02 (-0.05 to 0.01)	0.22	-0.02 (-0.05 to 0.01)	0.25
HTN*Systolic SD	-0.001 (-0.05 to 0.05)	0.95	0.006 (-0.04 to 0.05)	0.80	0.005 (-0.04 to 0.05)	0.83
HTN*Diastolic SD	-0.04 (-0.12 to 0.04)	0.34	0.006 (-0.06 to 0.08)	0.86	0.007 (-0.06 to 0.08)	0.84
HTN*Systolic COV	0.04 (-0.03 to 0.11)	0.25	0.02 (-0.04 to 0.08)	0.47	0.02 (-0.04 to 0.08)	0.52
HTN*Diastolic COV	-0.006 (-0.07 to 0.05)	0.85	0.01 (-0.04 to 0.06)	0.67	0.01 (-0.04 to 0.07)	0.65
HTN*Systolic VIM	0.06 (0.01 to 0.11)	0.01	0.02 (-0.02 to 0.07)	0.25	0.02 (-0.02 to 0.07)	0.29
HTN*Diastolic VIM	-0.01 (-0.09 to 0.07)	0.78	0.01 (-0.06 to 0.08)	0.68	0.02 (-0.05 to 0.08)	0.67

Table S5. Association between blood pressure variability and cerebral arterial dilation adjusted for BMI.

ORI Adjusted Model 2 was the initial multivariable model adjusted for age, sex and vascular risk factors including cigarette use, alcohol consumption, diabetes, hyperlipidemia, hypertension and history of cardiovascular disease. NEW Adjusted Model was additionally adjusted for BMI which included 2249 individuals with BMI records. BP, blood pressure. SD, standard deviation. COV, coefficient of variation. VIM, variation independent of mean. MCA, middle cerebral artery.

*General linear model with logistic link function and binomial distribution was used for the binary response variable.

	ORI Adjusted Model 2 (n=2634)		NEW bivariable Model (n=2249)		NEW Adjusted Model 2(n=2249)	
	β (95% CI)	P value	β (95% CI)	P value	β (95% CI)	P value
Diameter of MCA						
Systolic BP	0.001 (-0.002 to 0.005)	0.55	8.8×10^{-4} (-0.002 to 0.004)	0.60	8.1×10^{-4} (-0.003 to 0.005)	0.67
Diastolic BP	-0.002 (-0.008 to 0.004)	0.53	-0.002 (-0.008 to 0.004)	0.47	-0.004 (-0.01 to 0.003)	0.30
Systolic SD	0.01 (0.001 to 0.02)	0.03	0.01 (0.002 to 0.02)	0.02	0.004 (-0.006 to 0.01)	0.41
Diastolic SD	0.001 (-0.01 to 0.02)	0.89	0.002 (-0.01 to 0.02)	0.82	-0.008 (-0.02 to 0.007)	0.29
Systolic COV	0.01 (0.002 to 0.03)	0.02	0.02 (0.003 to 0.03)	0.01	0.006 (-0.007 to 0.02)	0.38
Diastolic COV	0.002 (-0.009 to 0.01)	0.72	0.003 (-0.009 to 0.01)	0.64	-0.005 (-0.02 to 0.007)	0.44
Systolic VIM	0.01 (7.4×10^{-4} to 0.02)	0.03	0.01 (0.002 to 0.02)	0.02	0.004 (-0.006 to 0.01)	0.40
Diastolic VIM	0.003 (-0.01 to 0.02)	0.74	0.003 (-0.01 to 0.02)	0.66	-0.006 (-0.02 to 0.009)	0.42
Dilation of MCA*						
Systolic BP	0.02 (0.003 to 0.04)	0.02	0.02 (-4.7×10^{-4} to 0.04)	0.06	0.02 (-0.005 to 0.04)	0.13
Diastolic BP	0.008 (-0.03 to 0.04)	0.65	-0.002 (-0.04 to 0.03)	0.91	-0.001 (-0.04 to 0.04)	0.94
Systolic SD	0.08 (0.04 to 0.12)	$P<0.001$	0.07 (0.02 to 0.11)	0.003	0.07 (0.02 to 0.11)	0.005
Diastolic SD	0.05 (-0.02 to 0.13)	0.15	0.03 (-0.05 to 0.11)	0.42	0.03 (-0.05 to 0.12)	0.45
Systolic COV	0.11 (0.05 to 0.17)	$P<0.001$	0.09 (0.03 to 0.16)	0.005	0.09 (0.03 to 0.16)	0.006
Diastolic COV	0.04 (-0.01 to 0.10)	0.14	0.03 (-0.03 to 0.09)	0.37	0.03 (-0.04 to 0.09)	0.41
Systolic VIM	0.07 (0.03 to 0.12)	0.001	0.06 (0.01 to 0.11)	0.01	0.07 (0.01 to 0.12)	0.01
Diastolic VIM	0.05 (-0.02 to 0.13)	0.14	0.04 (-0.04 to 0.12)	0.37	0.03 (-0.05 to 0.12)	0.41

Table S6. Association between blood pressure variability and cerebral arterial dilation in hypertension individuals.

Model 1 adjusted for age and sex; Model 2 adjusted for age, sex and vascular risk factors including cigarette use, alcohol consumption, diabetes, hyperlipidemia and history of cardiovascular disease. BP, blood pressure. SD, standard deviation. COV, coefficient of variation. VIM, variation independent of mean. MCA, middle cerebral artery.

*General linear model with logistic link function and binomial distribution was used for the binary response variable.

	Bivariable model		Adjusted Model 1		Adjusted Model 2	
	β (95% CI)	P value	β (95% CI)	P value	β (95% CI)	P value
Diameter of MCA						
Systolic BP	7.2×10^{-4} (-0.003 to 0.005)	0.74	4.3×10^{-4} (-0.004 to 0.005)	0.84	3.5×10^{-4} (-0.004 to 0.005)	0.87
Diastolic BP	-0.002 (-0.009 to 0.006)	0.67	-0.003 (-0.01 to 0.005)	0.51	-0.002 (-0.010 to 0.006)	0.64
Systolic SD	0.02 (0.004 to 0.03)	0.006	0.01 (0.003 to 0.02)	0.01	0.01 (0.003 to 0.02)	0.01
Diastolic SD	5.6×10^{-4} (-0.02 to 0.02)	0.95	-0.002 (-0.02 to 0.02)	0.85	-6.6×10^{-4} (-0.02 to 0.02)	0.94
Systolic COV	0.02 (0.007 to 0.04)	0.004	0.02 (0.005 to 0.04)	0.01	0.02 (0.005 to 0.04)	0.01
Diastolic COV	0.001 (-0.01 to 0.02)	0.84	-1.7×10^{-4} (-0.01 to 0.01)	0.98	3.8×10^{-4} (-0.01 to 0.01)	0.96
Systolic VIM	0.02 (0.006 to 0.03)	0.004	0.02 (0.004 to 0.03)	0.009	0.02 (0.004 to 0.03)	0.01
Diastolic VIM	0.002 (-0.02 to 0.02)	0.86	-4.0×10^{-4} (-0.02 to 0.02)	0.97	3.5×10^{-4} (-0.02 to 0.02)	0.97
Dilation of MCA*						
Systolic BP	0.02 (-1.5×10^{-4} to 0.04)	0.052	0.02 (-0.002 to 0.04)	0.07	0.02 (-0.004 to 0.04)	0.12
Diastolic BP	4.9×10^{-4} (-0.04 to 0.04)	0.98	0.006 (-0.03 to 0.05)	0.75	0.01 (-0.03 to 0.05)	0.61
Systolic SD	0.10 (0.06 to 0.15)	*P*<0.001	0.10 (0.05 to 0.14)	*P*<0.001	0.10 (0.05 to 0.14)	*P*<0.001
Diastolic SD	0.05 (-0.04 to 0.13)	0.27	0.04 (-0.04 to 0.13)	0.35	0.04 (-0.04 to 0.13)	0.32
Systolic COV	0.16 (0.09 to 0.22)	*P*<0.001	0.15 (0.08 to 0.22)	*P*<0.001	0.15 (0.08 to 0.22)	*P*<0.001
Diastolic COV	0.04 (-0.03 to 0.10)	0.26	0.03 (-0.04 to 0.10)	0.37	0.03 (-0.04 to 0.10)	0.37
Systolic VIM	0.12 (0.07 to 0.17)	*P*<0.001	0.11 (0.06 to 0.17)	*P*<0.001	0.11 (0.06 to 0.17)	*P*<0.001
Diastolic VIM	0.05 (-0.04 to 0.14)	0.26	0.04 (-0.05 to 0.13)	0.36	0.04 (-0.05 to 0.13)	0.36

Table S7. Association between blood pressure variability and cerebral arterial dilation in non-hypertension individuals.

Model 1 adjusted for age and sex; Model 2 adjusted for age, sex and vascular risk factors including cigarette use, alcohol consumption, diabetes, hyperlipidemia and history of cardiovascular disease. BP, blood pressure. SD, standard deviation. COV, coefficient of variation. VIM, variation independent of mean. MCA, middle cerebral artery.

*General linear model with logistic link function and binomial distribution was used for the binary response variable.

	Bivariable model		Adjusted Model 1		Adjusted Model 2	
	β (95% CI)	P value	β (95% CI)	P value	β (95% CI)	P value
Diameter of MCA						
Systolic BP	0.004 (-0.003 to 0.01)	0.27	0.003 (-0.004 to 0.010)	0.39	0.003 (-0.003 to 0.010)	0.34
Diastolic BP	-0.002 (-0.01 to 0.009)	0.74	-0.003 (-0.01 to 0.009)	0.66	-0.002 (-0.01 to 0.009)	0.68
Systolic SD	0.003 (-0.01 to 0.02)	0.73	0.002 (-0.01 to 0.02)	0.85	0.002 (-0.01 to 0.02)	0.82
Diastolic SD	0.006 (-0.02 to 0.03)	0.65	0.004 (-0.02 to 0.03)	0.75	0.005 (-0.02 to 0.03)	0.73
Systolic COV	0.003 (-0.02 to 0.02)	0.78	0.002 (-0.02 to 0.02)	0.87	0.002 (-0.02 to 0.02)	0.86
Diastolic COV	0.006 (-0.01 to 0.03)	0.54	0.005 (-0.01 to 0.02)	0.62	0.005 (-0.01 to 0.02)	0.6
Systolic VIM	0.001 (-0.01 to 0.02)	0.89	6.6×10^{-4} (-0.01 to 0.02)	0.93	6.8×10^{-4} (-0.01 to 0.02)	0.93
Diastolic VIM	0.008 (-0.02 to 0.03)	0.55	0.006 (-0.02 to 0.03)	0.63	0.007 (-0.02 to 0.03)	0.61
Dilation of MCA*						
Systolic BP	0.04 (0.01 to 0.08)	0.006	0.04 (0.003 to 0.07)	0.03	0.03 (-1.9×10^{-4} to 0.07)	0.051
Diastolic BP	-0.02 (-0.08 to 0.04)	0.57	-0.005 (-0.07 to 0.06)	0.89	-0.007 (-0.07 to 0.06)	0.83
Systolic SD	0.05 (-0.03 to 0.13)	0.26	0.03 (-0.05 to 0.12)	0.43	0.03 (-0.05 to 0.12)	0.43
Diastolic SD	0.09 (-0.04 to 0.22)	0.18	0.07 (-0.06 to 0.20)	0.31	0.07 (-0.06 to 0.20)	0.32
Systolic COV	0.04 (-0.07 to 0.14)	0.52	0.02 (-0.09 to 0.14)	0.68	0.03 (-0.09 to 0.14)	0.64
Diastolic COV	0.08 (-0.02 to 0.17)	0.11	0.06 (-0.04 to 0.15)	0.24	0.06 (-0.04 to 0.15)	0.24
Systolic VIM	0.002 (-0.08 to 0.08)	0.96	-9.0×10^{-4} (-0.08 to 0.08)	0.98	0.002 (-0.08 to 0.08)	0.95
Diastolic VIM	0.10 (-0.02 to 0.23)	0.11	0.07 (-0.05 to 0.20)	0.25	0.07 (-0.05 to 0.20)	0.25

Table S8. Interaction between blood pressure variability and hypertension on the association of cerebral arterial dilation.

Model 1 adjusted for age and sex; Model 2 adjusted for age, sex and vascular risk factors including cigarette use, alcohol consumption, diabetes, hyperlipidemia and history of cardiovascular disease. BP, blood pressure. SD, standard deviation. COV, coefficient of variation. VIM, variation independent of mean. MCA, middle cerebral artery.

*General linear model with logistic link function and binomial distribution was used for the binary response variable.

	Bivariable model		Adjusted Model 1		Adjusted Model 2	
	β (95% CI)	P value	β (95% CI)	P value	β (95% CI)	P value
Diameter of MCA						
HTN*Systolic BP	-0.004 (-0.01 to 0.004)	0.35	-0.003 (-0.01 to 0.005)	0.49	-0.003 (-0.01 to 0.005)	0.50
HTN*Diastolic BP	-9.0×10^{-4} (-0.01 to 0.01)	0.89	-3.6×10^{-4} (-0.01 to 0.01)	0.96	5.3×10^{-4} (-0.01 to 0.01)	0.94
HTN*Systolic SD	0.008 (-0.01 to 0.03)	0.41	0.008 (-0.01 to 0.03)	0.40	0.008 (-0.01 to 0.03)	0.40
HTN*Diastolic SD	-0.007 (-0.04 to 0.02)	0.67	-0.007 (-0.04 to 0.03)	0.69	-0.005 (-0.04 to 0.03)	0.74
HTN*Systolic COV	0.01 (-0.01 to 0.04)	0.29	0.01 (-0.01 to 0.04)	0.31	0.01 (-0.01 to 0.04)	0.31
HTN*Diastolic COV	-0.006 (-0.03 to 0.02)	0.65	-0.006 (-0.03 to 0.02)	0.64	-0.005 (-0.03 to 0.02)	0.67
HTN*Systolic VIM	0.01 (-0.006 to 0.03)	0.18	0.01 (-0.007 to 0.03)	0.23	0.01 (-0.007 to 0.03)	0.23
HTN*Diastolic VIM	-0.007 (-0.04 to 0.02)	0.65	-0.007 (-0.04 to 0.02)	0.64	-0.007 (-0.04 to 0.02)	0.68
Dilation of MCA*						
HTN*Systolic BP	-0.03 (-0.06 to 0.01)	0.18	-0.02 (-0.06 to 0.02)	0.30	-0.02 (-0.05 to 0.02)	0.40
HTN*Diastolic BP	0.02 (-0.05 to 0.09)	0.63	0.03 (-0.05 to 0.10)	0.48	0.03 (-0.04 to 0.10)	0.40
HTN*Systolic SD	0.04 (-0.05 to 0.13)	0.41	0.04 (-0.05 to 0.13)	0.41	0.04 (-0.05 to 0.13)	0.38
HTN*Diastolic SD	-0.04 (-0.20 to 0.11)	0.59	-0.03 (-0.19 to 0.12)	0.66	-0.03 (-0.18 to 0.13)	0.74
HTN*Systolic COV	0.09 (-0.03 to 0.22)	0.14	0.09 (-0.04 to 0.21)	0.17	0.09 (-0.04 to 0.21)	0.18
HTN*Diastolic COV	-0.04 (-0.15 to 0.07)	0.49	-0.04 (-0.15 to 0.08)	0.52	-0.03 (-0.15 to 0.08)	0.56
HTN*Systolic VIM	0.10 (0.004 to 0.19)	0.041	0.09 (-0.006 to 0.18)	0.07	0.09 (-0.009 to 0.18)	0.08
HTN*Diastolic VIM	-0.05 (-0.20 to 0.10)	0.50	-0.05 (-0.20 to 0.10)	0.53	-0.04 (-0.19 to 0.11)	0.58

Figure S1. Scheme of manual determination of cerebral vessels and Representative manually drawn points.

A, Scheme of manual determination the M1 segment of middle cerebral artery. B, M1 segment of middle cerebral artery.

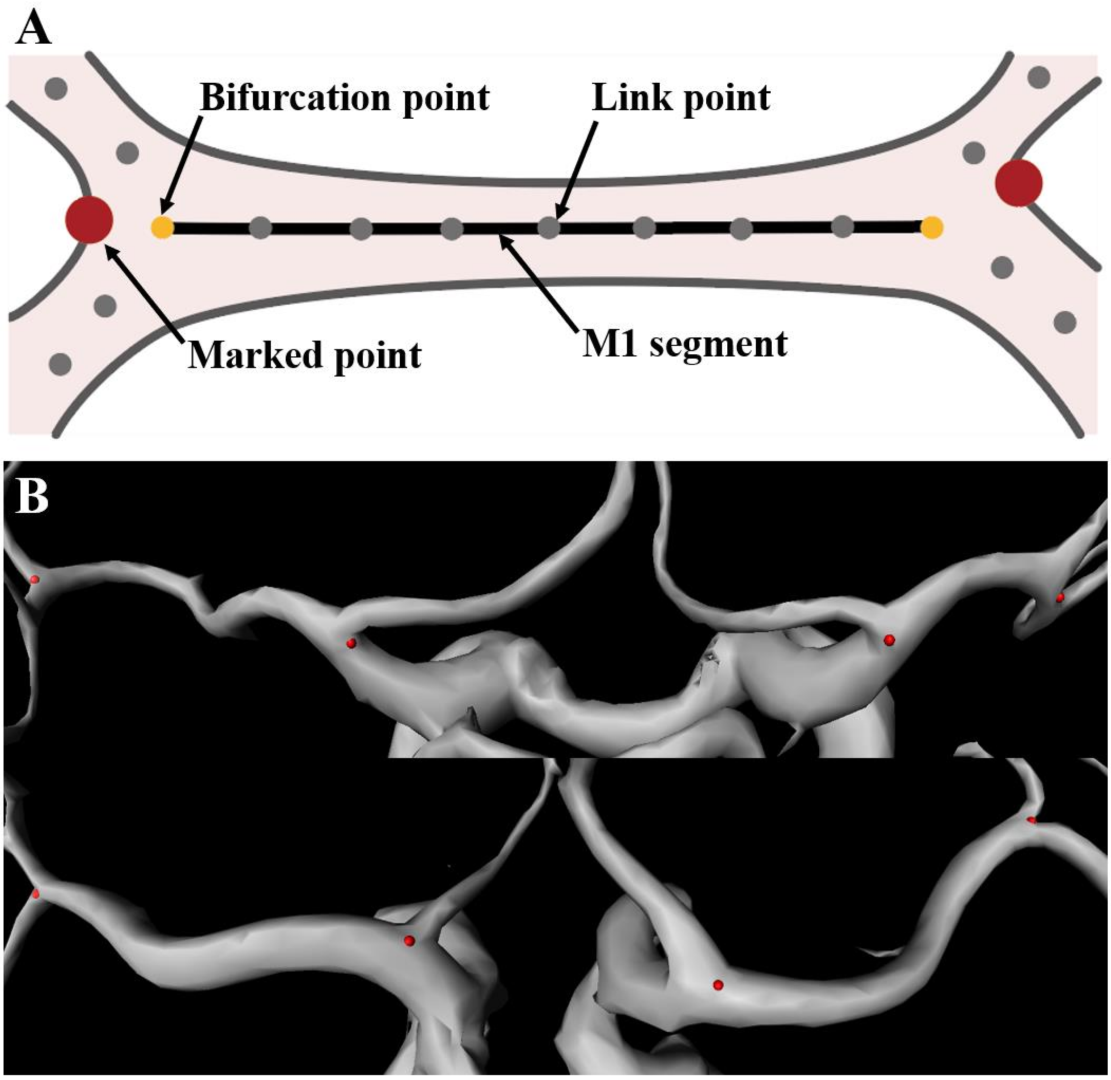

Figure S2 Blood pressure and variability associated distribution of white matter hyperintensity.

A, systolic blood pressure. B, diastolic blood pressure. C, SD of systolic blood pressure. D, SD of diastolic blood pressure. E, COV of systolic blood pressure. F, COV of diastolic blood pressure. SD, standard deviation. COV, coefficient of variation. VIM, variation independent of mean.

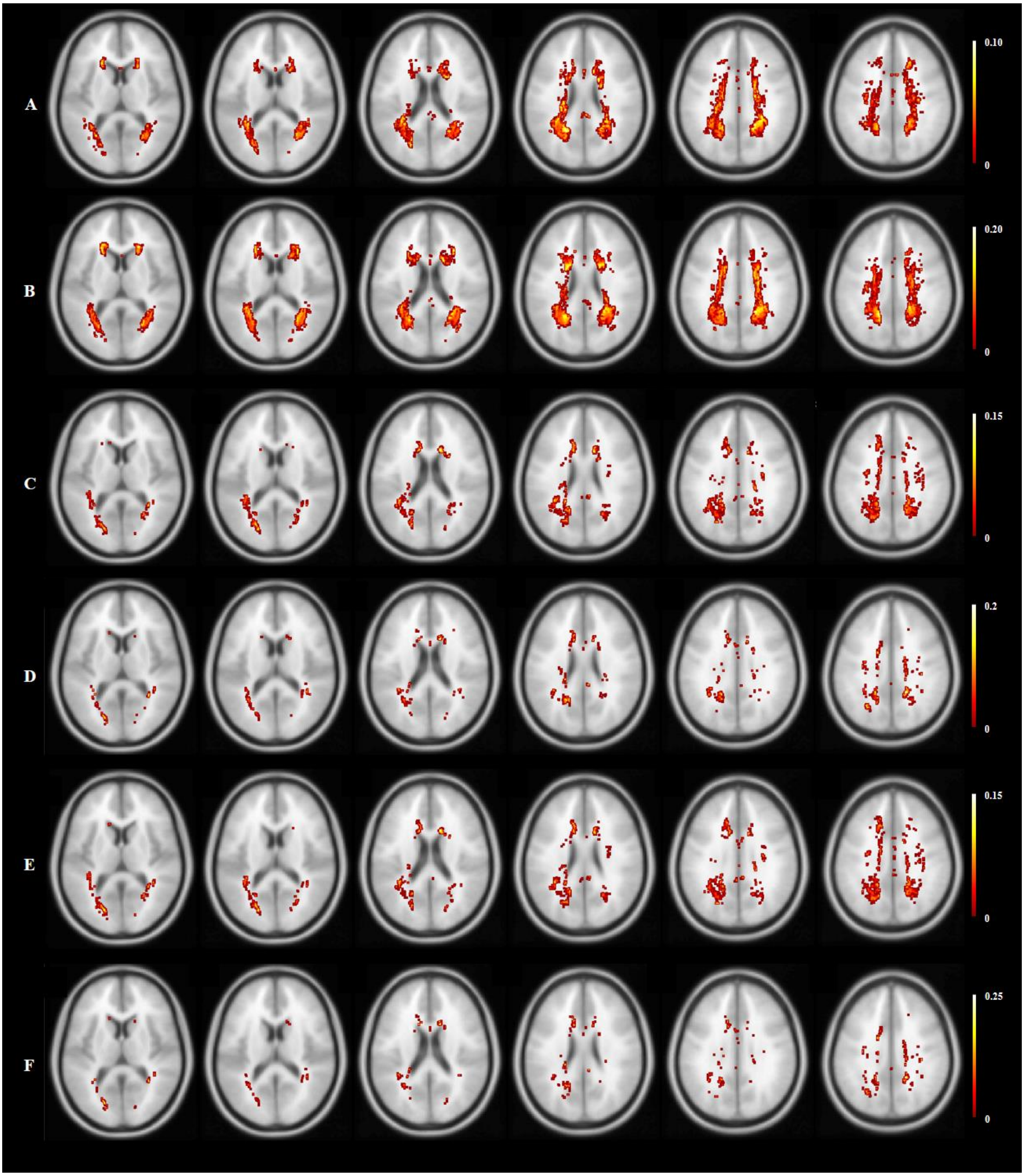

Supplement: Supplementary file 1 [file hyp-79-1455-s001.pdf]
